# Advances in Human-Computer Interactions: Methods, Algorithms, and Applications

**DOI:** 10.1155/2018/4127475

**Published:** 2018-07-05

**Authors:** Fabio Solari, Manuela Chessa, Eris Chinellato, Jean-Pierre Bresciani

**Affiliations:** ^1^University of Genoa, Genoa, Italy; ^2^Middlesex University, London, UK; ^3^University of Fribourg, Fribourg, Switzerland

The recent prevalence of new technologies and devices for the fruition of multimedia contents (e.g., head-mounted-displays, augmented reality devices, smartphones and tablets) has been changing the modality of accessing and exploring the digital information, by introducing novel human-computer interactions (HCIs) modalities. The even growing market demand, on the one hand, has pushed the diffusion of such technologies; on the other hand, it has hampered a detailed analysis of their effects on the users. In particular, perceptual evidence from cognitive sciences and neurosciences has to be considered during the design of HCI systems in order to decrease visual fatigue and cybersickness and to lead to natural HCI in virtual and augmented reality (VR/AR) environments. The use of physiological signal, such as electroencephalography (EEG), can lead to more effective multimodal interfaces that, from one hand, allow people to better handle the VR environment and, on the other hand, allow the system to anticipate the user's actions. Such systems allow us to design VR environment that can be used in medical applications. Moreover, computer science and artificial intelligence can provide techniques to design systems that adapt themselves to the specific characteristics of each user by producing personalized interfaces that allow a natural HCI, by taking into account the sensorimotor control aspects that arise by using such systems.

The articles contained in the present issue include research articles as well as review articles with a focus on cognitive aspects and computational intelligence techniques to improve the HCI systems in order to obtain natural and ecological ways to interact with digital contents in VR and AR environments

The contribution by L.M. Alonso-Valerdi and V.R. Mercado-García, “Enrichment of Human-Computer Interaction in Brain-Computer Interfaces via Virtual Environments,” provides an extensive review of recent advances and future perspectives of the use of Virtual Reality for improving human-computer interaction in highly demanding and interactive systems, such as brain-computer interfaces.

In the paper by B. Binias et al., “A machine learning approach to the detection of pilot's reaction to unexpected events based on EEG signals,” the authors discuss the existing neural network techniques to discriminate between states of brain activity related to idle but focused anticipation of visual cue and reaction to it by using electroencephalographic signals for cognitive cockpits.

HCI and VR technologies represent nowadays a popular solution for physical rehabilitation and motor control research. E. D. Oña et al., in their paper “Effectiveness of Serious Games for Leap Motion on the Functionality of the Upper Limb in Parkinson's Disease: A Feasibility Study,” propose and discuss the design and application of Serious Games based on the Leap Motion sensor, as a tool to support the rehabilitation therapies for upper limbs. In particular, they assess the therapeutic effectiveness of the proposed system, by defining a protocol of trials with Parkinson's patients. Their results are encouraging and go in the direction of an effective use of VR in clinical practice.

Sensory aspects of HCI are investigated in “Recurrent Transformation of Prior Knowledge Based Model for Human Motion Recognition,” by C. Xu et al. The authors have developed a novel technique for human activity recognition using wearable sensor data based on the addition of preliminary conceptual knowledge to a decision tree classifier. Experimental validation shows that the proposed methodology is able to outperform alternative state of the art methods.

Several EEG-based brain-computer interface systems rely on Steady-State Visually Evoked Potentials (SSVEP). In their contribution entitled “Sinc-Windowing and Multiple Correlation Coefficients Improve SSVEP Recognition Based on Canonical Correlation Analysis,” V. Mondini and colleagues propose an approach based on a slightly modified Canonical Correlation Analysis to improve the accuracy of the classification algorithm.

In their contribution entitled “Analysis of User Interaction with a Brain-Computer Interface Based on Steady-State Visually Evoked Potentials: Case Study of a Game,” H. M. de Arruda Leite and colleagues used a computer game as case study to evaluate different aspects of a brain-computer interface (BCI). The game consisted of using the BCI to move a ball on a board to collect coins. This study allowed the authors to identify some pitfalls and the overall results are quite promising.

The contribution of this special issue is of presenting studies on HCI improvements that can attract attention by the scientific community to pursue further investigations leading to VR systems that can be effectively used in real world situations.

